# Isolated Femoral LCL Avulsion Fracture in the Adult Knee: Two Patient Cases and Literature Review

**DOI:** 10.1155/2022/6507577

**Published:** 2022-04-20

**Authors:** Gilles Dietrich, Benoît Maeder, John Nyland, Yaël Maeder, Alain Akiki, Robin Martin

**Affiliations:** ^1^Orthopaedic Department, Lausanne University Hospital, Lausanne, Switzerland; ^2^Orthopaedic Department, Hôpital Riviera-Chablais, Rennaz, Switzerland; ^3^Athletic Training Program, Kosair Charities College of Health and Natural Sciences, Spalding University, USA; ^4^Radiology Department, Lausanne University Hospital, Lausanne, Switzerland

## Abstract

Avulsion injuries of the LCL most commonly occur at the fibular insertion. Femoral LCL avulsion injuries have only been previously described in pediatric patients or as multiligament knee injury components among adults. This case series with comprehensive literature review describes for the first time 2 cases of isolated LCL femoral avulsion fractures in adults including conservative treatment outcomes. Both patients sustained a strong varus stress mechanism to their right knee, following sport injury or road traffic accident. For both patients, a complete radiographic evaluation including X-rays, MRI, and CT scan confirmed no other associated knee lesions. The femoral LCL avulsion fractures that were observed were minimally displaced and noncomminuted. Furthermore, imagery suggested preserved integrity at the superior lateral genicular artery, adjacent articular capsule, and IT band. Based on clinical and imaging evaluations, the decision was made to follow conservative treatment. By 10 weeks postinjury, both patients were asymptomatic with early radiological fracture healing evidence. Comparative varus stress radiographs at 20° knee flexion revealed no side-to-side differences and clinical exam showed no posterolateral rotatory instability. The second patient case presented with mild femoral LCL attachment calcification on follow-up CT-scan. Following a detailed analysis of anatomic injury characteristics, we suggest that patients with isolated femoral LCL avulsion fractures have low secondary displacement risk provided SLGA, articular capsule, and IT band integrity are present. In contrast to high-grade ligamentous and distal avulsion LCL injuries, we recommend conservative treatment for patients who sustain these lesions.

## 1. Introduction

Among sport-related injuries, knee injuries are most common accounting for 39% of those according to epidemiological studies [1], reaching as high as 73.9% [2]. The most commonly injured knee structures are the medial collateral ligament (MCL), the patellar tendon, the anterior cruciate ligament (ACL), and menisci [1, 3–5]. Studies agree that lateral collateral ligament (LCL) injuries are far less common, representing only 1.1% of all knee injuries [1]. Therefore, it is difficult to draw conclusions regarding which life activities and sports present the greatest injury risk.

A large, retrospective Swiss study reported that soccer (football) and skiing accounted for the largest number of knee injuries, but tennis and gymnastics involved the largest number of LCL injuries [1]. In contrast to MCL injury [6], the high forces that produce LCL injuries are more often associated with concomitant ACL or posterior cruciate ligament (PCL) injury, leading to significant functional impairments [7–10]. Isolated LCL injuries more frequently involve lower magnitude forces, resulting in less ligament damage; therefore, high-grade isolated LCL injuries are rare [11]. The LCL, popliteus tendon, popliteofibular ligament, and knee joint capsule are the primary posterolateral corner (PLC) components that stabilize the lateral knee [12, 13]. The LCL is important for resisting varus loads at all degrees of knee flexion and for restraining tibial external rotation and posterior translation when the knee is extended [13, 14]. The proximal LCL attachment is located immediately posterior to the lateral femoral epicondyle, with some fibers extending in a fan-like pattern proximally and anteriorly over the epicondyle [15]. The superior lateral genicular artery (SLGA) is located above this LCL attachment. The SLGA provides vascularisation to all lateral femoral condyle region tissues, including the LCL attachment [16].

Complete LCL tears (grade III injury) most commonly occur at the fibular insertion (44.4%) followed by the midsubstance (33.3%) and the femoral insertion (22.3%) [11]. Regarding LCL avulsion fractures, most involve the fibular attachment, warranting surgical intervention [17].

Few isolated femoral LCL avulsion fracture injuries have been reported in the literature. A single-patient case study identified femoral LCL avulsion fracture in association with PLC injury [18]. Another study reported 14 femoral LCL avulsion cases; however, delineation between soft tissue and bony attachment injury site locations was not appreciable [19]. Less than 10 cases have been reported among skeletally immature patients [20, 21]. The purpose of this case report and literature review was to describe the treatment outcomes of two patients with distinctly different isolated femoral LCL avulsion fracture injuries. Information from this report should provide a useful reference for physicians who treat these injuries.

### 1.1. Case 1

A 21-year-old male mountain biker, in otherwise good health and without previous knee injury, consulted our emergency department because of lateral side, right knee pain following a high-velocity crash. The injury mechanism consisted of the inside of his right thigh colliding with a tree trunk generating a pure varus knee stress. He was unable to ambulate immediately following the event.

On clinical examination, he presented tenderness to palpation over the lateral femoral epicondyle of the right knee. There was no appreciable knee effusion. Anterior-posterior drawer and the Lachman Tests were negative; however, both active and passive knee range of motion (ROM) were severely impaired. There was no evidence of neurovascular impairment. The patient presented no general knee laxity and had a no obvious varus malalignment.

**Figure 1:**
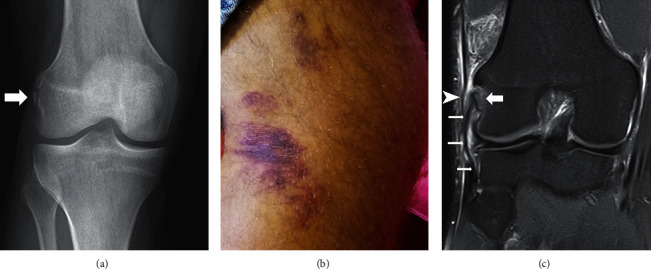
Patient case 1. (a) AP right knee X-ray at day 1 showing the avulsion fracture of the femoral insertion of the LCL (white arrow). (b) Clinical appearance at 2 weeks showing bruises over lateral aspect of the right knee. (c) Proton density coronal MRI of the right knee at day 3 showing a wavy though intact LCL (small arrows) attached to the avulsed bony fragment of the femoral insertion (large arrow). The iliotibial band (arrowhead) lies immediately superficial to the proximal LCL.

Standard radiographs revealed bony avulsion from the lateral femoral epicondyle. Magnetic resonance imaging (MRI) revealed an isolated bony fragment of 11 mm (AP) × 4 mm (ML) × 13 mm (PD) in size, which was minimally displaced (3 mm) from its bed **(**Figure 1**)**. The LCL, the popliteus tendon, the iliotibial band (IT band), and the SLGA were intact. The anterolateral ligament could not be identified on the MRI in this patient. There was no other associated lesion.

Based on clinical and imaging evaluations, the decision was made to treat the patient with conservative fracture management through use of a hinged knee brace to prevent the varus stresses that would load the LCL. Over the initial 3 weeks postinjury, the knee brace was locked in full extension. Following this, the brace was unlocked to allow up to 30° knee flexion over the ensuing 2 weeks. Thereafter, knee flexion ROM was increased 30° at 2-week intervals until full ROM was achieved. Weight bearing as tolerated crutch ambulation was allowed over the entire treatment course. Localized ecchymosis developed at the lateral knee over the first 3 weeks of treatment; however, lateral knee pain gradually decreased with each follow-up visit to a visual analog scale (VAS) pain score of 0.

### 1.2. Case 2

A 28-year-old cook, in otherwise good health and without significant medical-surgical history was involved in a high-velocity road traffic accident when his lower limbs were struck from behind by a car at bilateral midlower leg level as he was standing facing the back of his car.

**Figure 2:**
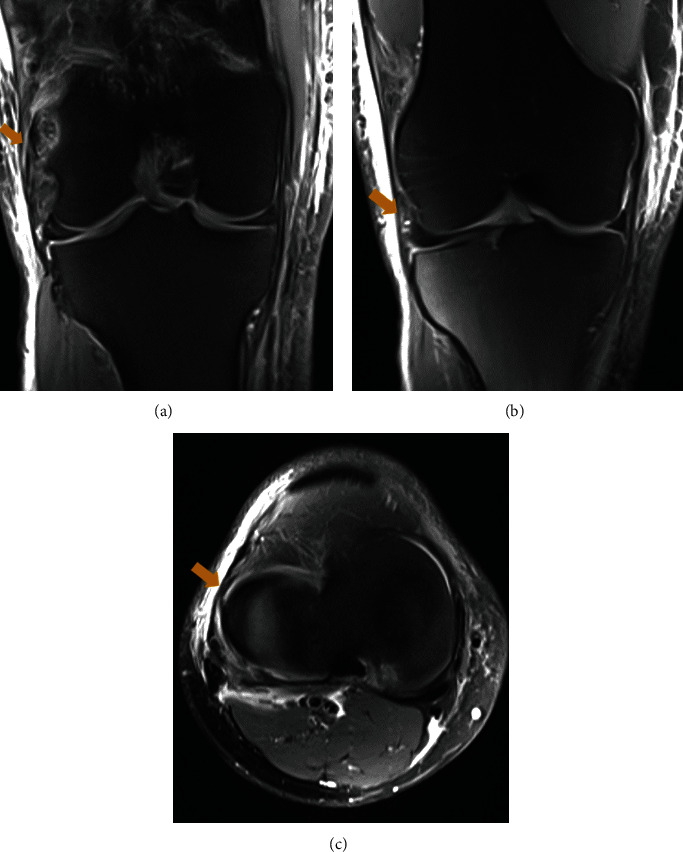
Patient case 2. (a, b, and c) Proton density coronal and axial MRI of the right knee at day 3 showing a loose though intact ALL (gold arrows).

**Figure 3:**
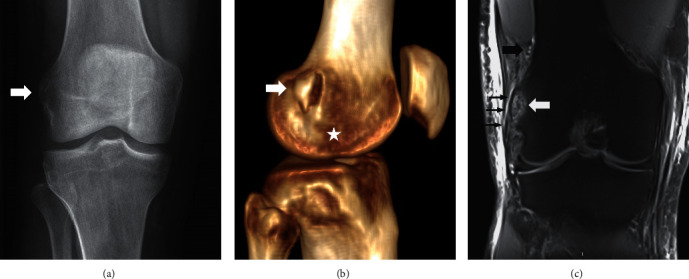
Patient case 2. (a) AP right knee X-ray at day 1 showing the avulsion fracture of the femoral insertion of the LCL (white arrow). (b) Lateral view of a 3D reconstruction of the right knee CT at day 1 showing the avulsed bony fragment of the femoral insertion of the LCL (white arrow). The white star shows insertion site of the popliteal tendon. (c) Proton density coronal MRI obtained at day 3 showing an intact LCL (small black arrows) attached to the avulsed bony fragment of the femoral insertion (large white arrow). Note the superior lateral genicular vessels (large black arrow) above the bony fragment.

The patient was treated at a trauma resuscitation room where whole-body CT scanning was performed. This revealed open fractures at the right (Gustilo-Anderson grade II) and left (Gustilo-Anderson grade IIIc) distal legs, with isolated LCL insertion avulsion at the right knee. Bony avulsion dimensions were 10 mm (AP) × 4 mm (ML) × 18(PD) mm with 1 mm displacement from its bed (1 mm). Right knee MRI confirmed that the LCL, popliteus tendon, IT band, and anterolateral ligament (ALL) were intact. However, the ALL appeared loose. This might result of a concomitant avulsion of the ALL with the LCL at their proximal insertion (Figure 2). There was no associated soft tissue or SLGA lesion (Figure 3).

Since massive tibia, fibular, and soft tissue loss made the left leg unsalvageable, the patient underwent a first-line below knee amputation (Burgess technique). The distal right leg was treated with open reduction and internal fixation using an anterolateral distal tibial plate (LCP Anterolateral Distal Tibia Plate 3.5, 21 holes, DePuy Synthes, Warsaw, IN, USA). Postsurgery, a hinged knee brace could not be used at the right lower extremity because the surgical repair was immobilized in a short leg cast. Over the initial 6 weeks postsurgery, full weight-bearing activities were limited to bed-to-chair transfers.

### 1.3. Radiographic Patient Case Comparisons

**Figure 4:**
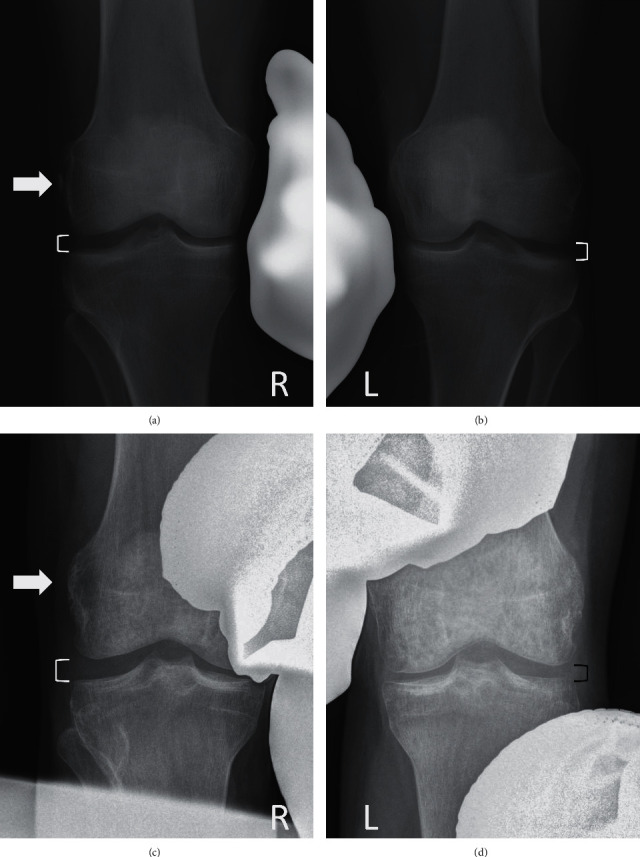
Comparative varus stress X-rays. (a and b) Patient case 1, at 14 months. Radiographic bone union of the fracture on the right knee (white arrow) as well as symmetrical side-to-side opening of the lateral compartment (white brackets). (c and d) Patient case 2, at 5 months. Radiographic bone union of the fracture on the right knee (white arrow) and 2 mm side-to-side difference between the right knee (white bracket) and the left knee (black bracket).

In the first patient case, radiographs obtained at 4 weeks postinjury failed to reveal bony fragment displacement. Repeat radiographs at 10 weeks postinjury displayed early fracture healing. At this time, the patient presented no knee instability with varus stress at 20° of flexion and full active ROM was restored. At this time, hinged knee brace use was discontinued. Comparative varus stress radiographs at 20° knee flexion were performed at 14 months postinjury, revealing an absence of side-to-side lateral joint space opening differences and radiological evidence of complete fracture healing (Figures 4(a) and 4(b)). The pivot sift test was negative.

**Figure 5:**
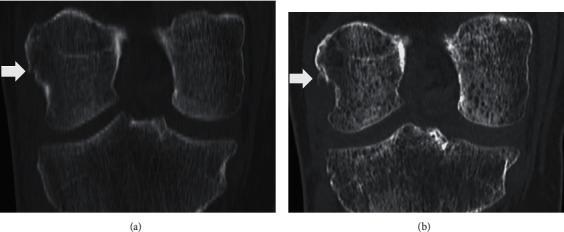
Patient case 2. Frontal plane CT assessment. (a) At day 1, showing the avulsed bony fragment of the femoral insertion of the LCL (white arrow). (b) At 4.5 months, showing bone union with mild calcification of the femoral attachment of the LCL (white arrow).

In the second patient case, radiographs taken at 3 weeks postsurgery failed to reveal bony fragment displacement and full-length hip-to-ankle radiographs demonstrated a right knee varus deformity of 6°. Comparative varus stress radiographs at 20° knee flexion revealed lateral tibiofemoral compartment gapping of approximately 2 mm greater than the opposite side **(**Figures 4(c) and 4(d)**)**. However, these views were considered suboptimal given postamputation stress test application limitations. Radiologic fracture healing evidence was also observed at this time. Mild calcification at the femoral LCL attachment was highlighted on follow-up CT-scan at 4.5 months postsurgery **(**Figure 5**)**. Clinically, the patient presented no instability with varus stress test at 20° of flexion and full active ROM was achieved. There was no posterolateral rotatory instability. The pivot shift test was negative.

## 2. Discussion

To our knowledge, no previous patient case of an isolated femoral LCL avulsion fracture in an adult has been reported. In fact, literature searches only identified a few cases of femoral LCL avulsion in adults with each occurring with other associated knee musculoligamentous injuries. One case report described femoral LCL avulsion fracture combined with complete PCL and popliteus tendon rupture. In this case, the fracture was managed surgically and the patient recovered full active knee ROM and could perform all activities of daily living [18]. Recently, Kahan et al. reported 14 cases of femoral LCL avulsions in a cohort of 100 patients who had experienced multiligament knee injuries; however, they did not delineate between soft tissue and bony detachment injuries [19].

Among pediatric patients, femoral LCL avulsion injuries have been described in several reports. In a Swedish study, 5 skeletally immature patients (<14 y/o) were diagnosed with this lesion; however, all were associated with concomitant femoral popliteus tendon avulsion [21]. Four of these patients were treated surgically and one conservatively. All four operated patients had a better treatment outcome than the nonoperated patient based on the Lysholm Knee Score and the KOOS subscale scores [21]. Kramer et al. reported about two children who had sustained femoral LCL avulsion fractures that were successfully managed conservatively; however, information about associated knee lesions or the rehabilitation strategy was not provided [20]. Therefore, we could not conservative treatment efficacy. Furthermore, neither of these studies provided fracture displacement measurements [20, 21].

Since 2013, our institution has been recording adult patient knee ligament injury and treatment outcome information in a registry. The 2 patient cases discussed in this paper represent two unique isolated femoral LCL avulsion fracture cases from this database, representing 0.03% of all new cases admitted over a 6-year period. Over the same period, we recorded 6.5 times more fibular LCL avulsion fractures representing 13 cases, or 0.19% of all new cases admitted over the same 6 year period. Similarly, Kahan et al. reported that fibular LCL avulsion injuries were 3.3 more frequent in their cohort of multiligament knee injuries (46 fibular vs. 13 femoral LCL avulsion injuries) [19]. In contrast, MCL avulsion fractures reportedly occur more frequently at the femoral attachment than at the tibial attachment [22, 23].

In this case report, we describe, for the first time, the characteristics and anatomical relationships of isolated femoral LCL avulsion fracture in adults. In both patients, the avulsed femoral fragment was isolated, minimally displaced, and noncomminuted. We surmise that it was partially stabilized by femoral LCL attachment expansion fibers, the intact lateral knee joint capsule, and the IT band, each of which lies immediately superficial to the proximal LCL. These structures likely help prevent secondary fracture displacement, despite the naturally unstable nature of the external femorotibial joint. Simultaneous avulsion of the ALL can occur, as illustrated in our second case. Although the precise origin of the ALL remains somewhat controversial, it has a close relation to the LCL proximal insertion [24] and previous authors had already reported concomitant proximal disruption of the ALL and LCL [25].

In contrast to femoral LCL avulsion fractures, the characteristics and anatomical relationships of fibular LCL avulsion fractures, also known as “arcuate fractures,” are well known. They have been often observed as comminuted proximal fibular head fractures involving the LCL, biceps femoris tendon, and popliteofibular ligament [17]. They may also be combined with IT band and capsular avulsions from the tibia with high secondary displacement risk. Secondary displacement risk for this fracture type is likely influenced by proximal biceps femoris tendon forces and posterior popliteofibular ligament forces. These lesions are located distal to the IT band and lateral knee joint capsule.

We hypothesized that the knee joint capsule which covers the proximal LCL provides important secondary stabilization following femoral LCL avulsion fracture. Knee joint capsule preservation in each presented case was related to an absence of hemarthrosis. Although intra-articular effusion was noted in both patient cases, it offered a CT density value of <30 Hounsfield units, which is too low for hemarthrosis. The low T1 signal and lack of fluid-fluid level on MRI/CT also supported the absence of hemarthrosis, suggesting joint capsule integrity. We were unable to directly confirm proximal knee joint capsule integrity, however, as it was inconspicuous due to extensive fracture region soft tissue edema [26, 27].

The periosteum of the femoral LCL attachment is perfused by the SLGA. This artery almost invariably originates from the popliteal artery. It then branches into a superior branch, which supplies the distal femoral shaft, the lateral femoral condyle (LFC) and the overlying soft tissues, and an inferior branch supplying the intercondylar fossa, knee capsule, and LFC. Wong et al. studied the SLGA LFC periosteal perfusion area reporting a distribution extending 12 cm proximally from the joint line, including the femoral LCL attachment [28]. Despite close proximity between the SLGA and femoral LCL avulsion fracture location, we failed to observe any MRI evidence of vascular injury in these 2 patient cases. The tissue healing recovery timeline would likely have been prolonged had the SLGA been disrupted.

Since the two convex articular surfaces of the lateral tibiofemoral joint are naturally unstable, high-grade LCL injuries often display residual instability following conservative treatment. In contrast, with a convex proximal joint surface over a concave distal joint surface, the medial tibiofemoral joint generally displays good conservative MCL injury treatment results, even when combined with complete internal ligament complex rupture [29–32]. Based on these osseous morphological characteristics, surgical treatment is generally favored for midsubstance grade III LCL injuries, whether isolated or in combination with multiligament knee injury [4, 8]. Surgical treatment is also favored for arcuate fractures since they are more frequently subject to primary or secondary displacement. Based on information obtained from a systematic review, Geeslin et al. recommended that to improve objective knee stability following fibular avulsion fractures associated with grade III posterolateral corner knee injuries, the avulsed structures should be anatomically repaired at the time of cruciate ligament reconstruction [17].

In contrast to grade III LCL lesions, we hypothesized that isolated femoral LCL avulsion fractures would benefit from conservative, nonoperative treatment. Conservative treatment should be considered if the fracture is minimally displaced, isolated, surrounded by a well-preserved knee joint capsule and adequately perfused by an intact SLGA. Since they serve as knee varus restraints, ACL, PCL, and posterolateral corner capsuloligamentous integrity are also essential determinants in the decision to select a conservative treatment approach [30]. Both patients discussed in this case report met these criteria, so we believed that conservative treatment would be successful.

In the first patient case, a hinged knee brace similar to the one used during rehabilitation for an isolated MCL injury was used [6]. In the second patient case, no brace was prescribed because weight bearing was not possible before 6 weeks. Since a similar positive conservative treatment outcome was observed with and without knee brace use, one may question its necessity for conservative treatment. We believe that hinged knee brace use is preferable to protect the knee whenever the patient has the potential to be ambulatory and at least partial weight bearing.

As described by Pellegrini and Stieda [33], MCL femoral avulsion fracture and proximal tears are known to promote adjacent soft tissue calcification. The second patient case displayed mild femoral LCL attachment calcification on follow-up CT-scan (Figure 4). This observation appears similar to that observed by Pellegrini-Stieda at the medial knee [33].

## 3. Conclusion

We report two patient cases of isolated femoral LCL avulsion fracture in the adult. Following a detailed analysis of anatomic injury characteristics, we suggest that these lesions are at low secondary displacement risk provided that they are surrounded by a well-preserved knee joint capsule and IT band. The tissue healing recovery timeline benefits from confirmation of an intact vascular supply from the SLGA. Based on these patient experiences, in contrast to high-grade ligamentous and distal avulsion LCL injuries, we recommend conservative treatment for these lesions.

## Data Availability

The data used to support the findings of this study are included within the article.

## Abbreviations

MCL: Medial collateral ligament
ACL: Anterior cruciate ligament
LCL: Lateral collateral ligament
PLC: Posterolateral corner
SLGA: Superior lateral genicular artery
ROM: Range of motion
MRI: Magnetic resonance imaging
CT: Computerized tomography scan
AP: Anterior-posterior
ML: Medial-lateral
PD: Proximal-distal
IT: Iliotibial
LFC: Lateral femoral condyle
ALL: Anterolateral ligament.
